# Deep learning for risk-based stratification of cognitively impaired individuals

**DOI:** 10.1016/j.isci.2023.107522

**Published:** 2023-08-02

**Authors:** Michael F. Romano, Xiao Zhou, Akshara R. Balachandra, Michalina F. Jadick, Shangran Qiu, Diya A. Nijhawan, Prajakta S. Joshi, Shariq Mohammad, Peter H. Lee, Maximilian J. Smith, Aaron B. Paul, Asim Z. Mian, Juan E. Small, Sang P. Chin, Rhoda Au, Vijaya B. Kolachalama

**Affiliations:** 1 Department of Medicine, Boston University Chobanian & Avedisian School of Medicine, Boston, MA, USA; 2 Department of Radiology and Biomedical Imaging, University of California, San Francisco, San Francisco, CA, USA; 3 Department of Computer Science, Boston University, Boston, MA, USA; 4 Department of Medicine, Stanford University School of Medicine, Stanford, CA, USA; 5 Department of Anatomy and Neurobiology, Boston University Chobanian & Avedisian School of Medicine, Boston, MA, USA; 6 Department of General Dentistry, Boston University School of Dental Medicine, Boston, MA, USA; 7 The Framingham Heart Study, Boston University Chobanian & Avedisian School of Medicine, Boston, MA, USA; 8 Department of Biostatistics, Boston University School of Public Health, Boston, MA, USA; 9 Department of Radiology, Lahey Hospital & Medical Center, Burlington, MA, USA; 10 Department of Radiology, Massachusetts General Hospital, Boston, MA, USA; 11 Department of Radiology, Boston University Chobanian & Avedisian School of Medicine, Boston, MA, USA; 12 Department of Brain and Cognitive Sciences, Massachusetts Institute of Technology, Cambridge, MA, USA; 13 Center of Mathematical Sciences & Applications, Harvard University, Cambridge, MA, USA; 14 Boston University Alzheimer’s Disease Research Center, Boston, MA, USA; 15 Department of Epidemiology, Boston University School of Public Health, Boston, MA, USA; 16 Department of Neurology, Boston University Chobanian & Avedisian School of Medicine, Boston, MA, USA; 17 Faculty of Computing & Data Sciences, Boston University, Boston, MA, USA

**Keywords:** Health sciences, Illness behavior

## Abstract

Quantifying the risk of progression to Alzheimer’s disease (AD) could help identify persons who could benefit from early interventions. We used data from the Alzheimer’s Disease Neuroimaging Initiative (ADNI, n = 544, discovery cohort) and the National Alzheimer’s Coordinating Center (NACC, n = 508, validation cohort), subdividing individuals with mild cognitive impairment (MCI) into risk groups based on cerebrospinal fluid amyloid-β levels and identifying differential gray matter patterns. We then created models that fused neural networks with survival analysis, trained using non-parcellated T1-weighted brain MRIs from ADNI data, to predict the trajectories of MCI to AD conversion within the NACC cohort (integrated Brier score: 0.192 [discovery], and 0.108 [validation]). Using modern interpretability techniques, we verified that regions important for model prediction are classically associated with AD. We confirmed AD diagnosis labels using postmortem data. We conclude that our framework provides a strategy for risk-based stratification of individuals with MCI and for identifying regions key for disease prognosis.

## Highlights

• We developed a deep learning model to stratify progression risk to Alzheimer’s disease
• We measured generalizability of the model in an external cohort
• We assessed alignment of Alzheimer’s disease pathology and clinical labels
• We found relevant brain regions that are important for predicting progression risk

## Graphical abstract

**Figure:**
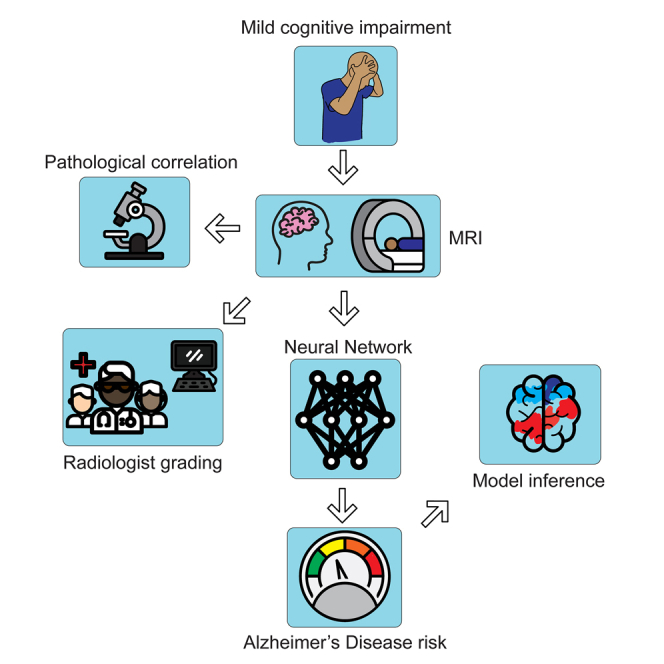

## Introduction

The projected cost of caring for millions of individuals who have Alzheimer’s disease (AD) worldwide is going to exceed a trillion dollars in a few years.^1^ In addition to the enormous health burden, patients and their caregivers experience financial, physical, and psychological strain. A theory regarding repeated drug failure in AD is that patients undergoing experimental therapies are selected too late in the disease process.^2^ Therefore, it is important to identify patients at a high risk of progression to AD in early stages of the disease. Further, as disease-modifying therapies are undergoing regulatory scrutiny,^3^ at-risk persons who are identified in a timely fashion could benefit from such interventions.

Not all individuals with mild cognitive impairment (MCI) develop AD.^4^ To this end, several frameworks have been constructed to identify individuals with normal cognition or MCI who progress to AD. The most common approach has been to use a classification framework to distinguish individuals who remain stable with MCI from those who progress to AD dementia.^5^^,^^6^^,^^7^^,^^8^ Most of these classifiers have demonstrated excellent performance, utilizing clinical data, demographic data, and/or imaging data in combination. Models have reached receiver-operating-characteristic areas-under-the-curve of over 0.9.^5^ Others have focused instead on building models to predict time-to-progression by estimating survival, or time-to-event, curves.^9^^,^^10^^,^^11^^,^^12^^,^^13^ These frameworks predominantly utilize a combination of clinical, biological, and imaging measurements to forecast disease progression. Performance of these models has commonly been assessed with concordance index (CI), which measures how well a model captures risk rank-ordering.^10^^,^^14^ The CI is a measurement that takes the predicted risk of some event for each sample in a dataset, and for all available pairs of samples, takes the proportion where the sample with the higher predicted event risk experiences the event earlier.^13^^,^^15^^,^^16^

Deep learning survival models specifically have been developed and utilized in several areas of medicine like oncology, and have demonstrated improved performance over more conventional survival analyses.^17^^,^^18^ Such models, in addition to conventional machine-learning models, have also been applied to forecast progression from MCI to AD.^12^^,^^13^^,^^19^^,^^20^ While certain deep learning survival frameworks such as DeepSurv^21^ utilize a Cox-proportional hazards-based model, there are several deep learning survival frameworks that are more flexible and do not rely on the proportional hazard assumption, such as neural multi-task regression, Weibull-based survival models, DeepHit, and Nnet-survival.^13^^,^^20^^,^^22^^,^^23^ The former three have demonstrated excellent performance in the setting of AD. However, attempts at utilizing MRI to directly model time-to-progression in AD have so far relied on data from pre-specified brain regions parcellated with curated atlases.^13^^,^^24^ Others have utilized deep learning to extract features from demarcated regions such as the hippocampus.^25^ Additionally, generalizability of these high-performing models remains unclear, as most of them have relied on data from a single cohort. Moreover, attempts to map independent clinical evaluations with clinicopathologic associations were not considered. Such connections with the reference standards can ground model predictions with biological evidence. To address these gaps, we developed a flexible deep learning survival framework that can directly use structural brain MRIs, without prior assumptions or region of interest (ROI) selection, to forecast disease progression from MCI to AD. We further confirmed our findings with data from an external cohort, interpretability analysis and gold-standard evidence.

We hypothesized that models combining flexible survival prediction, such as Nnet-survival,^22^ with deep neural networks—either multilayer perceptrons (MLPs) or convolutional neural networks (CNNs)—and T1-weighted MRI would be more accurate in predicting progression risk than a Cox proportional hazard-based model. Cox proportional hazard-based models carry several assumptions, including the assumption that risk curves for different individuals differ by a constant multiple across time (i.e., the proportional hazard assumption).^16^^,^^26^ We hypothesized that a model that allowed for time-varying differences between samples instead could be of more value. We further hypothesized that, by utilizing a CNN with minimally processed MRIs as input, one can learn the imaging features necessary to make accurate predictions.

## Results

### Demographic analysis

**Figure 1:**
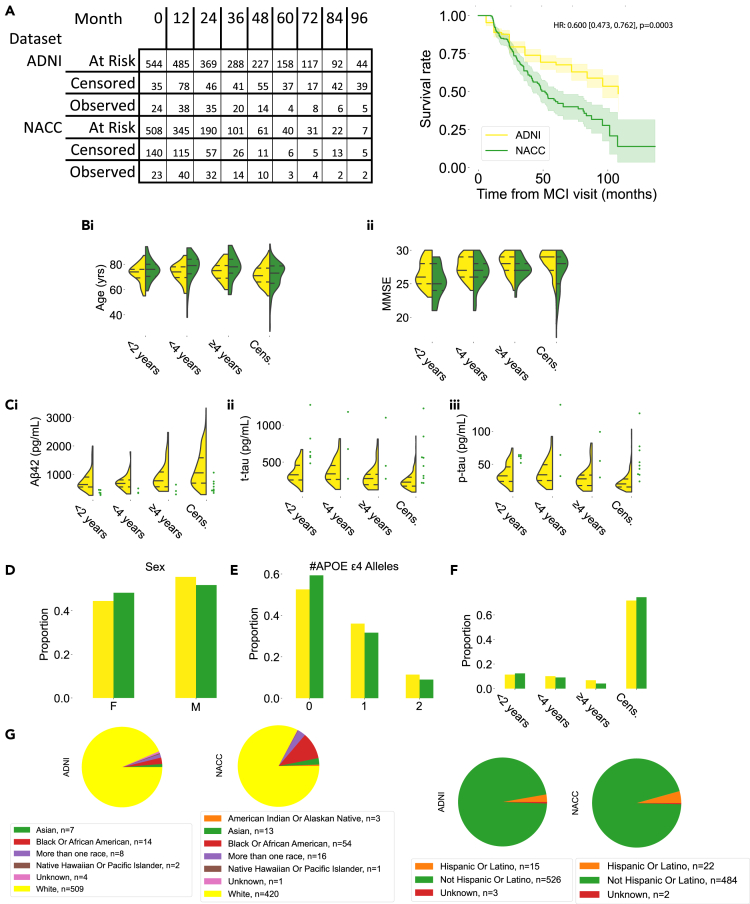
Study population Summary statistics of clinical and demographic parameters of persons from the Alzheimer’s Disease Neuroimaging Initiative (ADNI) and National Alzheimer's Coordinating Center (NACC) cohorts are shown. (A) Kaplan-Meier survival curves with 95% confidence intervals were computed for our two populations (ADNI: n = 544, 390 right-censored; NACC: n = 508, 378 right-censored). The number of persons at risk of progression, the number of persons censored, and the number of persons with “events”, or progression to AD, left-inclusive, are shown in the table to the left of the survival curves. The hazard ratio of the two curves is included. (B) The distribution of age (i) and Mini-Mental State Exam score (ii) for persons in the NACC and ADNI datasets are shown for persons in each progression category∗. (C) Concentration profiles of three different CSF biomarkers in the two cohorts, Aβ-42, total tau (t-tau), and phosphorylated tau (p-tau) are shown for persons in each progression category. Statistics were not computed for the NACC dataset due to the large amount of missing data. (D, E, and F) Distribution of sex and number of APOE e4 alleles of persons in each progression category, and the proportions of patients in each progression category are shown. (G) Pie charts summarizing the breakdown of race (left) and ethnicity (right) for each cohort. ∗Progression categories are—progression within 2 years, between 2 and 4 years, after 4 years, and censored or otherwise not progressed. See also Tables S1 and S2 for summary statistics for subplots 1A–1F.

We first characterized our discovery and validation cohorts (Figure 1; Tables S1 and S2). We found that persons in the National Alzheimer’s Coordinating Center (NACC) cohort progressed to AD more rapidly than those in the Alzheimer’s Disease Neuroimaging Initiative (ADNI) cohort beginning at 48 months (Figure 1A) (X^2^(1) = 0.06, p = 0.80 at 24 months; X^2^(1) = 14.56, p = 0.0001 at 48 months; n_NACC_ = 508, n_ADNI_ = 544 persons). The average age in the NACC cohort was higher than that of the ADNI cohort (Figure 1Bi) (Mann-Whitney U test, U = 127436, p = 0.029, n_NACC_ = 508, n_ADNI_ = 544). Overall, the ADNI participants received more education than NACC participants (Mann-Whitney U test, U = 153880, n_ADNI_ = 544, n_NACC_ = 508, p = 0.001). Mini-Mental State Exam (MMSE) scores are shown for reference in Figure 1Bii, though given the large number of NACC patients without concurrent MMSE scores at the time of MCI diagnosis, the discovery and validation datasets were not compared statistically.

CSF tau and p-tau levels were lower in persons who took more than 4 years to progress to AD (Figure 1C) (t-tau, Kruskal-Wallis test, H = 71.0, df = 3, p < 0.0001, n_<2 years_ = 390, n_<4 years_ = 55, n_>4 years_ = 37, n_Censored_ = 62; ≥ 4 years vs. < 2 years, p = 0.011, ≥4 years vs. < 4 years, 0.0086; p-tau, H = 79.4, df = 3, p < 0.0001, n_<2 years_ = 390, n_<4 years_ = 55, n_>4 years_ = 37, n_Censored_ = 62; ≥ 4 years vs. < 2 years, p = 0.011, ≥4 years vs. < 4 years, 0.0077; Benjamini-Hochberg-corrected p values for 6 pairwise comparisons). There were no statistically significant differences in sex (X^2^[3] = 1.33, p = 0.25, n_ADNI_ = 544, n_NACC_ = 508) or APOE e4 status (X^2^[3] = 4.37, p = 0.11, n_ADNI_ = 544, n_NACC_ = 379; 129 subjects in NACC without APOE data [Table S1; Figures 1D and 1E]). Proportions of persons in each of the progression groups used in other subplots of Figure 1 are shown in Figure 1F. Finally, ADNI had a larger proportion of persons identifying as “white” than NACC (Figure 1G) (X^2^[1] = 30.2, p < 0.0001, n_ADNI_ = 532, n_NACC_ = 491, with 12 ADNI and 17 NACC having an unknown race or identifying as multiple races). The two populations were not significantly different with respect to the proportion of persons identifying as Hispanic or Latino (X^2^[1] = 1.47, p = 0.23, n_ADNI_ = 541, n_NACC_ = 506, with 3 ADNI and 2 NACC patients having an unknown ethnicity).

### Survival-based validation of risk groups

**Figure 2:**
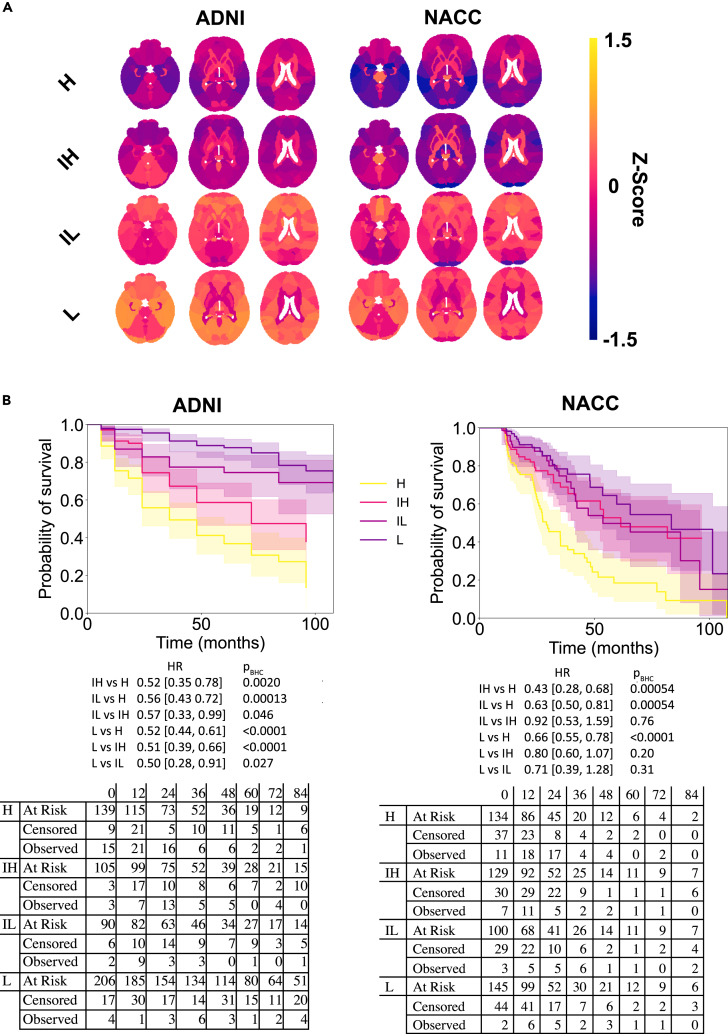
Distribution of risk-based groups (A) Heatmaps of gray matter volumes (GMVs) Z-scored to the mean and standard deviations of each region in the complete ADNI dataset (n = 544) are illustrated for persons in each risk group. Warmer colors indicate larger Z-scored GMVs and cooler colors indicate smaller Z-scored GMVs. (B) Survival curves for persons in each of the risk groups in the ADNI and NACC dataset, compared at time points 24, 48, and 96 months, with their 95% confidence intervals. The numbers of patients at risk of progression, the numbers censored, and the number that have progressed, left-inclusive, are shown below the survival curves, in addition to pairwise hazard ratios and their 95% confidence intervals. Benjamini-Hochberg-corrected p-values are included next to their corresponding pairwise hazard ratios. Comprehensive, pairwise statistics are shown in Table S3. H – high-risk; IH – intermediate-high-risk; IL – intermediate-low-risk; L – low-risk.

We sought to establish that we were still able to risk stratify our external dataset using anatomical features despite the differences between it and the discovery dataset. We divided persons in our discovery cohort by Aβ quartiles, then computed Z-scored gray matter volumes (GMVs) for persons within each quartile and assigned risk groups based on correlations with averaged Z-scored GMVs for each Aβ quartile. We correlated Z-scored GMVs in our external dataset with the averaged, Z-scored GMVs from the discovery dataset (Figure 2A). As expected, Z-scored GMVs within each of the four risk groups were highly correlated between the two cohorts (Spearman’s correlation coefficient, H in ADNI vs. H in NACC, ρ = 0.85, p < 0.0001; IH in ADNI vs. IH in NACC, ρ = 0.73, p < 0.0001; IL in ADNI vs. IL in NACC, ρ = 0.70, p < 0.0001; L in ADNI vs. L in NACC, ρ = 0.87, p < 0.0001; n = 66 brain regions for all comparisons). Additionally, Z-scored GMVs were lower overall in the higher-risk groups (Kruskal-Wallis, X^2^[3] = 158, p < 0.0001; H vs. IL, p < 0.0001; H vs. L, p < 0.0001; IH vs. IL, p < 0.0001; IH vs. L, p < 0.0001; H vs. IH, p = 0.83 and L vs. IL, p = 0.043; p values Benjamini-Hochberg corrected; n_H_ = 139, n_IH_ = 105, n_IL_ = 90, n_L_ = 206, ADNI dataset).

To confirm that differences in GMVs corresponded to differences in progression risk, we examined survival curves for each group in ADNI and NACC at different time points (Figure 2B, and Table S3). In the ADNI dataset, H persons had a significantly greater risk of AD progression compared with IH persons at times 24 months and 48 months (X^2^[1] = 7.02, p = 0.010, X^2^[1] = 4.84, p = 0.028, Benjamini-Hochberg corrected within each time and cohort) after MCI diagnosis. IH and IL persons differed at 48 months (X^2^[1] = 5.59, p = 0.027). Finally, IL and L persons had significantly different progression risk profiles at both 24 months and 48 months (X^2^[1] = 10.0, p = 0.0023; X^2^[1] = 4.81, p = 0.028). A similar pattern was evident in the external dataset. Specifically, H persons had a greater probability of progression than both IL and L persons at 24 months (X^2^[1] = 7.50, p = 0.018, X^2^[1] = 9.85, p = 0.010), and had a greater probability than L persons at 96 months (X^2^[1] = 8.45, p = 0.016). H persons also had a greater probability of progression than IH persons at 48 (X^2^[1] = 9.49, p = 0.006) and 96 months (X^2^[1] = 7.79, p = 0.016).

### Radiologist validation of atrophy patterns in different risk groups

**Figure 3:**
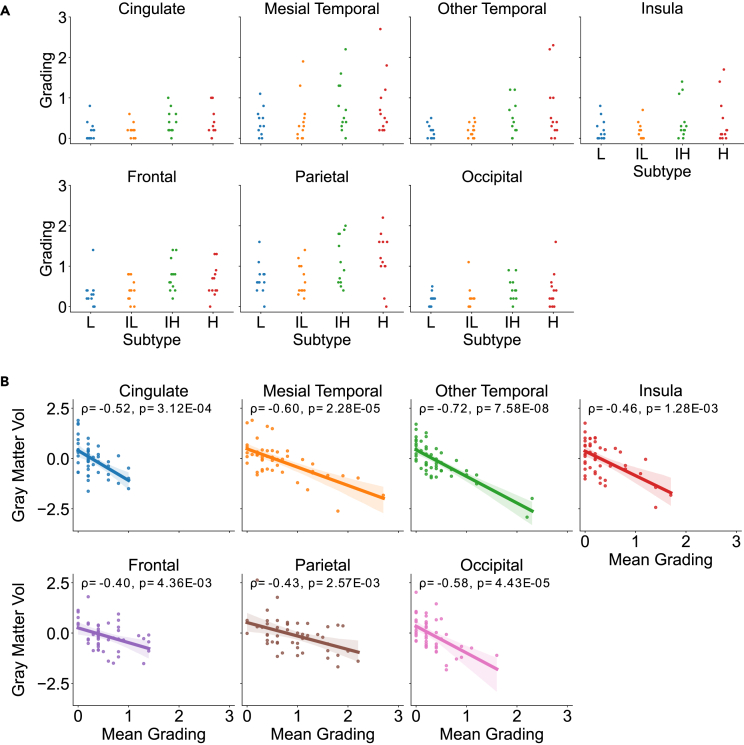
Radiologist confirmation of atrophy grade differences between groups (A) Radiologist atrophy grades for each of 7 brain regions that were reviewed, averaged across both hemispheres and across 5 different radiologists. The grade of atrophy in each region is denoted on the y axis, where 3 corresponds to “severe” atrophy, 2 to “moderate” atrophy, 1 to “mild” atrophy, and 0 to no atrophy. Grades are divided by subjects within the H, IH, IL, and L risk groups on the x axis. (B) Mean Z-scored GMVs of parcellated brain regions within each lobe are plotted against the mean radiologist’s grade within each graded lobe, averaged across hemispheres, with a 95% bootstrapped confidence interval using 1000 repetitions. Spearman correlation coefficients between mean Z-scored GMVs and radiologist grades are included within each plot, along with their Benjamini-Hochberg-corrected p-values.

Clinical validation of brain atlas-derived anatomical changes was performed with expert grading of 48 randomly sampled MRIs from ADNI, 12 from each risk group, and shown in Figure 3A. While agreement between radiologists was modest (mean ICC across all seven brain regions and hemispheres, 0.36 ± 0.13 (±sd), n_subjects_ = 48, n_raters_ = 5), their overall assessments revealed higher atrophy in the higher-risk groups (H and IH) compared with the lower risk groups (L and IL) in the noted regions aside from the insula (Mann Whitney-U test, U_Cingulate_ = 397, p = 0.026; U_Frontal_ = 432, p = 0.0084; U_Insula_ = 367, p = 0.10; U_Mesial Temporal_ = 399, p = 0.026; U_Occipital_ = 421, p = 0.0084; U_Other Temporal_ = 432, p = 0.0084; U_Parietal_ = 429, p = 0.0084 [Benjamini-Hochberg corrected p values, n_higher-risk_ = 24 averaged grades, n_lower-risk_ = 24 averaged grades for each comparison]) (Figure 3A).

Having observed our posited relationship between global atrophy and risk shown via expert grading, we next sought to confirm that our parcellation method captured regional atrophy. We found significant negative correlations between mean expert-grades for each lobe and mean Z-scored GMVs averaged over each lobe computed via our segmentation pipeline (Figure 3B), suggesting good correspondence between model-derived GMV estimates and brain atrophy.

### Deep learning models

**Figure 4:**
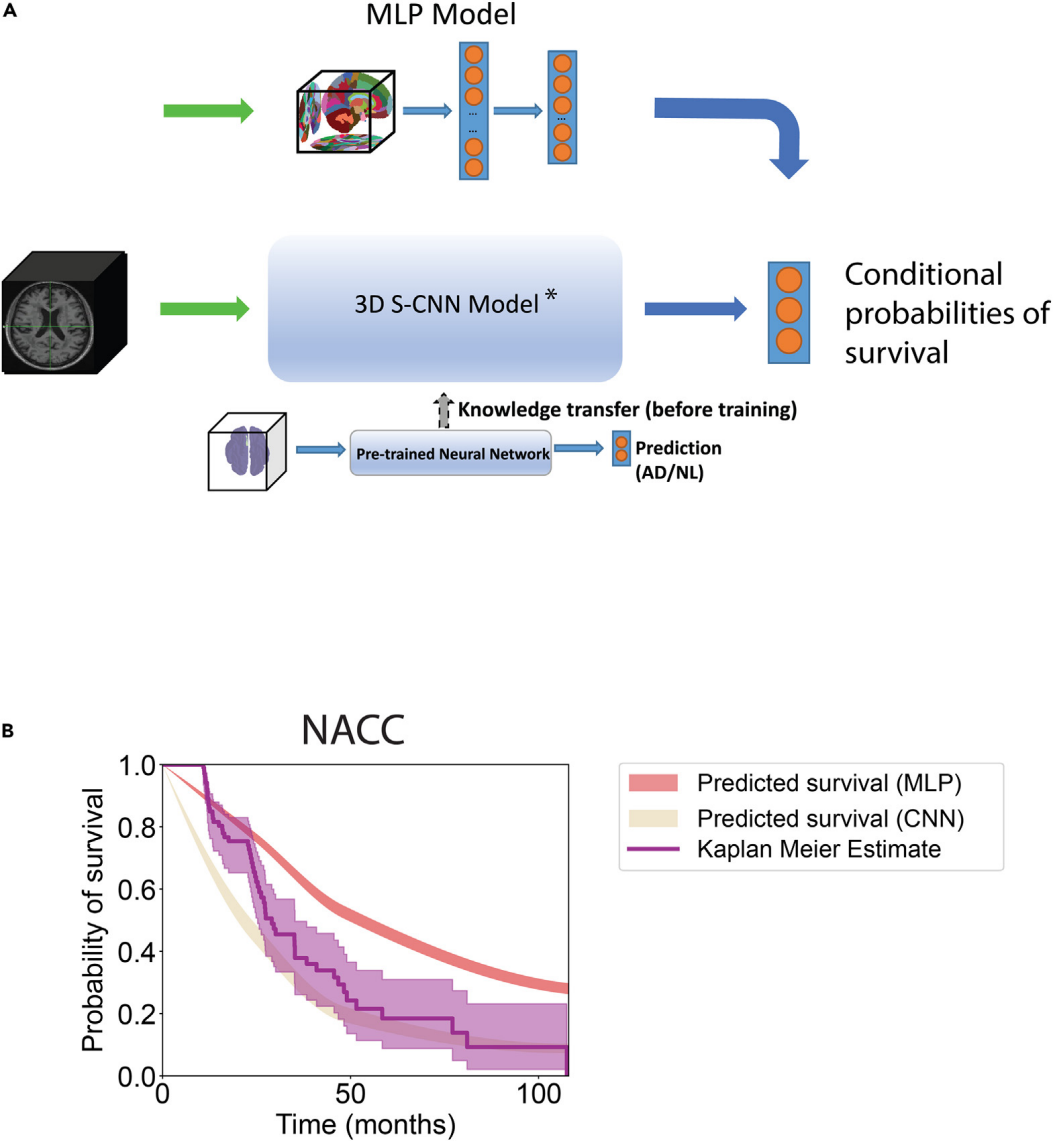
Schematics of the deep learning frameworks (A) Internal structure of a multilayer perceptron (MLP). Segmented GMVs were used as input to an MLP with two fully connected layers and used to predict the conditional probability of survival up to 24, 48, and 108 months. An S-CNN was also constructed to predict the same output. (B) An example comparison of empirical survival curves (Kaplan-Meier estimate with its 95% confidence interval) and predicted survival curves (interpolated in 1-month increments) using the conditional probabilities of survival from our MLP and S-CNN. Also, 95% confidence intervals around of the mean of predicted survival curves were computed via bootstrapping with 10,000 repetitions are shown. Here, “∗” indicates survival convolutional neural network, which can be seen in detail in Figure S1.

Once establishing the association of GMV atrophy with a biological measure of disease (CSF Aβ levels), and accordingly with progression risk to AD, we sought to generate a method of risk stratification that did not require *a priori* knowledge of CSF data, parameters such as a fixed number of risk groups, or parcellated brain regions. We trained multiple deep learning models, including baseline multi-layer perceptrons (MLPs) with and without CSF data, and a convolutional neural network utilizing a survival loss function (S-CNN), in addition to two reference models from the literature: an MLP utilizing a Weibull survival model (“Weibull”)^13^ and a baseline Cox proportional hazard (CPH) model combined with L1 and L2 loss. A general schematic for our models is shown in Figure 4A, with an exemplar prediction curve in Figure 4B and aschematic of our S-CNN in Figure S1. Models were evaluated and compared using concordance indices (CI) and integrated Brier scores (IBS). Higher CI and lower IBS correlate with increased risk-discrimination accuracy and calibration of probabilistic risk prediction models. While many studies investigating risk prediction in AD compare model performance using CI, few assess the magnitude of the difference between true versus predicted risk with metrics such as Brier scores. Given differences between Kaplan-Meier curves for NACC and ADNI (Figure 1A), we thought it was important to perform this step.

As seen in Table 1, all models performed comparably on the discovery dataset in terms of CI and IBS. Our MLPs did not differ in performance compared to the Weibull model in terms of CI and IBS on the validation dataset (MLP [GMV] vs. Weibull, t = 1.57, p = 0.21 for CI, t = 0.66, p = 0.61 for IBS; MLP [GMV + CSF], t = −0.39, p = 0.72 for CI, t = −2.16, p = 0.176 for IBS; all n = 5-folds). The highest performing S-CNN model on our validation dataset utilized transfer learning and fixed layers, demonstrating a CI of 0.676 ± 0.003 and integrated Brier score of 0.122 ± 0.013 (Table 1). Its CI was 4.4% lower than our MLP and 2.9% lower than the Weibull model (t = −8.88, p = 0.0044 vs. MLP; t = 3.40, p = 0.039 vs. Weibull model; corrected p values with Benjamini-Hochberg; n = 5-folds). The S-CNN did not differ significantly from the other models in IBS (t = 2.09, p = 0.176 vs. MLP [GMV]; t = 2.10, p = 0.176 vs. Weibull; t = −2.13, p = 0.176 vs. CPH; corrected p values with Benjamini-Hochberg; n = 5-folds for all comparisons).

**Table 1:** Model summaries and metrics

	ADNI		NACC	
	Concordance index	Integrated Brier score	Concordance index	Integrated Brier score
S-CNN	0 · 756 (0 · 051)	0 · 209 (0 · 050)	0 · 676 (0 · 003)^a^	0 · 122 (0 · 013)
MLP, GMV	0 · 731 (0 · 032)	0 · 192 (0 · 057)	0 · 707 (0 · 009)^b^	0 · 108 (0 · 010)
Weibull	0 · 743 (0 · 020)	0 · 205 (0 · 049)	0 · 696 (0 · 010)^b^	0 · 105 (0 · 005)
CPH	0 · 750 (0 · 051)	0 · 182 (0 · 055)	0 · 735 (0 · 014)^a^	0 · 104 (0 · 006)
MLP, GMV + CSF	0 · 729 (0.022)	0 · 181 (0 · 044)	0 · 699 (0 · 013)^b^	0 · 114 (0 · 008)

Mean concordance indices and integrated Brier scores for each of the listed models are shown. Metrics were averaged over 5-fold cross validation with standard deviation shown in parentheses. Concordance indices were averaged over the 3 time bins (24 months, 48 months, 108 months) for each fold. The MLPs were either trained with gray matter volumes from Neuromorphometrics parcellations, averaged over hemispheres [GMV], or with gray matter volumes and CSF volumes from Neuromorphometrics parcellations [GMV + CSF].

a Pairwise paired t-test comparisons revealed significant differences, after Benjamini-Hochberg correction, in model performance between CPH and the other models as well as between CNN and the other models.

b MLP [GMV], MLP [GMV + CSF], and Weibull models had larger Concordance Indices in the NACC dataset compared with the S-CNN after pairwise paired t-test comparisons, p values with Benjamini-Hochberg correction. Abbreviations: S-CNN – survival convolutional neural network; MLP – multilayer perceptron; CPH – Cox proportional hazard model.

### Importance of brain regions stratified by risk

**Figure 5:**
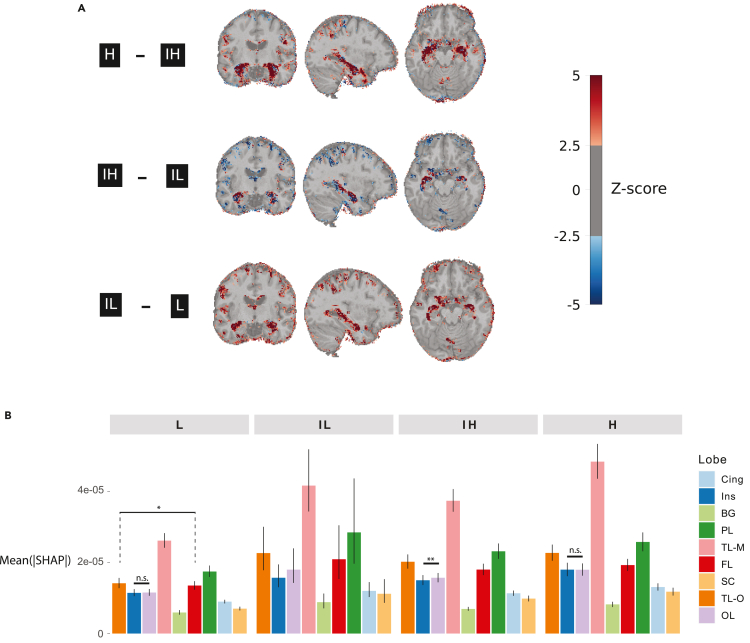
Cortical importance stratified by CSF-based risk groups (A) Differences between risk groups in **absolute** SHAP values averaged across all the time bins (24, 48, and 108 months), model predictions, and within each risk group for each voxel, computed for the external, NACC dataset. Shown in shades of blue are all voxels with a *Z* score of less than −2.0 and shown in shades of red are all voxels with a *Z* score of greater than 2.0. Z-scores were computed across all voxels for each subtracted brain. Values are overlayed on an exemplar subject’s pre-processed T1-weighted MRI. (B) Bar groups denoting the mean, absolute SHAP value for voxels in each cortical region, averaged for each participant in the NACC dataset. Error bars denote bootstrapped 95% confidence intervals. Here, n.s. = not significant; “∗” indicates p value < 0.05; “∗∗” indicates p value < 0.01; otherwise, all pairwise comparisons within each group p < 0.001.

We next investigated whether our CNN-based deep learning framework would reveal regions “important” for forecasting survival, whether important regions would differ based on CSF-driven risk group, and whether these would align with the literature. In organizing our results by CSF-driven risk group, we hoped to capture key differentiating features in persons with GMVs that appear broadly related to a biological correlate of AD. We computed SHAP values for our S-CNN model, given its comparable IBS to other state-of-the-art models. SHAP values with large magnitudes indicate that a particular voxel carries a large importance and portends either a significantly lower risk or higher risk of progression to AD in our model. Differences in importance of voxels between different CSF-driven risk groups are shown in Figure 5A. Most conspicuous here are voxels within the temporal lobe, suggesting that temporal lobe importance is a key differentiator between risk groups.

Averaged over voxels within each lobe, we see that the mesial temporal lobe has the largest importance across all risk groups (Figure 5B) (all pairwise p < 0.001, Wilcoxon signed-rank test, Benjamini-Hochberg corrected, n_H_ = 134, n_IH_ = 129, n_IL_ = 100, n_L_ = 145, Figure 5B). The parietal lobe and other regions of the temporal lobe demonstrate the next highest importance across all risk groups, followed by the frontal lobe (all p < 0.0001 aside from TL-O vs. FL in the low-risk group, p = 0.024, signed-rank sum = 4140, Wilcoxon signed-rank test, Benjamini-Hochberg corrected p values).

### Pathological confirmation of out-of-sample dataset labels

**Figure 6:**
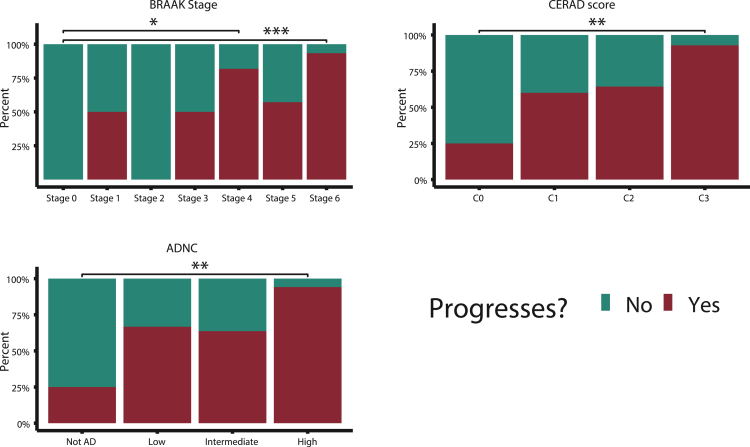
Risk group-specific associations with postmortem pathology The proportion of persons who progressed to AD versus those who remained stable with MCI are shown with respect to postmortem AD pathology measures ADNC, CERAD, and Braak staging. Pairwise Fisher exact test with Benjamini-Hochberg correction were used to evaluate for statistical significance in differences in proportion of persons who progressed with respect to severity of AD pathology measures. Here “∗” indicates p value < 0.05, “∗∗” indicates p value < 0.01, and “∗∗∗” indicates p value < 0.001.

Finally, in our external dataset, we sought to confirm accuracy of AD clinical diagnosis labels with postmortem pathology data (Figure 6). Clinical diagnoses are more readily available and used to train and test most models available in the literature. However, given that pathology is required to definitively confirm a diagnosis of AD,^27^ we wanted to confirm the accuracy of clinical diagnosis where possible.

Our NACC cohort contained 39 persons with ADNC grading, 46 persons with Braak staging, and 45 persons with CERAD scores. Figure 6 illustrates the proportion of persons who progress to AD versus those who remained MCI for each AD pathology measure; greater severity of AD pathology at autopsy was associated with clinically assessed AD progression. For each type of pathology, the proportion of individuals who were clinically determined to have progressed to AD was significantly higher for those in the most severe category (Braak Stage 6, CERAD C3, High ADNC) compared with the least severe category (Braak Stage 0, CERAD C0, Not AD) (pairwise Fisher exact tests, Benjamini-Hochberg correction; Braak: p = 0.001; CERAD: p = 0.0047; ADNC: p = 0.0061).

## Discussion

In this work, we demonstrate that (1) regional GMV correlates with Aβ levels and risk of progression from MCI to AD, (2) both MLPs and S-CNNs utilizing only structural imaging data in conjunction with a flexible survival loss function predict progression risk, and (3) our S-CNN model output appears to be driven by regions that we classically associate with AD pathology. Thus, these findings represent innovation at the intersection of neurology and computer science, while underscoring model conformity with biological evidence using routinely collected information such as structural MRI to quantify risk of progression from MCI to AD.

Most efforts to forecast MCI to AD progression have focused on performing a classification task, discriminating between persons who progress from MCI to AD and those who remain stable.^5^^,^^28^^,^^29^^,^^30^ Such models have achieved AUCs over 0.90 utilizing raw brain imaging, image-derived features, and/or clinical data.^5^ Other studies have attempted to measure risk utilizing hippocampal features from structural MRI,^12^ polygenic risk scores,^31^ or deep-learning derived image features.^32^ Some of these attempts to predict AD progression risk directly model survival curves utilizing survival methods incorporating imaging features as input.^13^^,^^16^^,^^20^ Only a handful of studies, though, have drawn connections between unparcellated structural imaging and disease progression.^26^^,^^33^^,^^34^ Further, none of these studies verified clinical labels with pathology.

As our two datasets, ADNI and NACC, exhibit different survival curves, direct comparison with many existing MCI to AD progression survival methods is challenging. While trained on a different task (predicting progression to subsequent stage of AD), N-MTLR boasted a C-index of 0.7781–0.7985 and an IBS between 0.0952 and 0.1086 utilizing only the NACC dataset.^20^ DeepHit-based and Weibull models have achieved concordance indices reaching 0.70–0.75 when forecasting MCI progression, albeit when trained and tested on a single dataset.^13^ Therefore, by selecting and implementing a state-of-the-art deep learning survival model using our data, which we denote as the Weibull model,^13^ we were able to both achieve a proper comparison to our models and establish external validation of another current state-of-the art model. Both of our MLPs and the Weibull model performed comparably on the discovery and validation datasets when using GMVs, though there was a drop-off in CI on the validation dataset. These models also had similar IBS on the validation dataset. Likely, the relative decrease in IBS from the discovery to validation dataset is an artifact of differences in censoring, as the weights used to compute IBS were calculated using the discovery cohort. Our S-CNN approach performed comparably to our MLP and to the Weibull model on internal validation and had 5.5–6.9% worse CI but similar IBS on the validation dataset. This constitutes an important contribution to the literature, as it provides a progression risk framework in which the model is allowed to discover salient features for itself.

A few other studies have focused on MCI progression using survival model frameworks with unparcellated structural imaging as input.^26^^,^^33^^,^^34^ ten Kate et al.^34^ used this approach to identify regions in which gray matter atrophy was highly predictive of progression from MCI to AD. Vemuri et al.^33^ similarly used a voxel-based approach to infer which brain regions are most predictive of progression. However, our deep learning approach performs this inference step while accomplishing the task of quantifying progression risk and validating this on an external dataset. While we can use our MLP, the Weibull model, or even our CPH model to forecast progression and perform inference, this limits us to inferences regarding only GMVs, for example. A model agnostic to this pre-processing step would be able to discover regions that are important for model predictions due to their white matter content, gray matter content, or more abstract content such as their spatial relationships to other regions. For example, in addition to identifying the importance of voxels within the most well-known AD-associated region, the mesial temporal lobe, our SHAP analysis suggests that voxels within the parietal lobe are also highly important for portending progression to AD in members from all risk subgroups. The parietal lobe is the region first affected in one of the four posited AD subtypes posited by Vogel et al.^35^ Given the growing calls within the machine learning community to evaluate the “black-box” nature of neural networks,^36^ our framework builds on our recent work in aiding interpretability,^37^^,^^38^ thereby grounding our results with established medical knowledge.

In summary, risk-based stratification could be critical to paving the way forward for physicians and pharmaceutical companies to provide targeted therapy for persons with MCI who would benefit from early interventions. We utilized survival-based deep neural networks in conjunction with minimally processed structural MRI, a widely available, non-invasive technique. Further, by employing state-of-the-art deep learning methods in conjunction with a SHAP analysis, we were able to identify regions particularly important for predicting increased progression risk. We submit that our practical approach to forecasting individualized progression risk in persons with MCI can have broad utility in various clinical and research settings with access to routinely collected structural neuroimaging data.

### Limitations of the study

There are a few limitations to our study. First, while we create a framework that estimates an individual’s risk of progression to AD based exclusively on structural MRI, we do not rely on demographic data such as years of education or on other imaging such as tau or amyloid-PET, diffusion imaging, or functional MRI. This is a strength in that our methodology does not require further input than a structural MRI to assess the risk of progression to AD, but also a weakness in that model performance could potentially be improved by incorporating these factors. For example, different patterns of tau deposition in AD have been found to correlate with rate of progression.^35^ Therefore, the addition of tau-PET data could potentially augment our risk-stratification framework, though availability of large, longitudinal, tau-PET datasets is limited. An additional limitation is that our survival analysis cannot model reversion from MCI to pre-MCI states; rather, we denote persons who have not developed AD after a diagnosis of MCI to be “non-converters.” While a different model could account for this, persons who revert from MCI have been shown to still be at higher risk than the average population of developing AD.^39^ Therefore, we believe that predicting progression to AD is still relevant for this population. Finally, we have a large amount of censoring in our datasets, with 378 persons not having an “event”, or AD progression, in our validation cohort, and 390 persons not having an “event” in the NACC cohort. The large amount of censoring in our dataset underscores the necessity of utilizing truly external datasets to validate results, as factors such as differences in loss to follow-up could potentially affect findings.

## STAR★Methods

### Key resources table

**Table:** 

REAGENT or RESOURCE	SOURCE	IDENTIFIER
**Biological samples**		

	ADNI dataset	RRID:SCR_003007
	NACC dataset	RRID:SCR_007327

**Software and algorithms**		

Computational Anatomy Toolbox (CAT) v12	Gaser et al.^40^	https://neuro-jena.github.io/cat/; RRID:SCR_019184
Neuromorphometrics atlas	Neuromorphometrics, Inc.	https://neuromorphometrics.com/2016-03/ProbAtlas.html; RRID:SCR_005656
Code from this paper	This paper	https://doi.org/10.5281/zenodo.8176269
SPM v12		https://www.fil.ion.ucl.ac.uk/spm/software/spm12/; RRID:SCR_007037
Weibull MLP	Nakagawa et al.^13^	https://doi.org/10.1093/braincomms/fcaa057
Survival loss function	Gensheimer and Narasimhan^22^	https://doi.org/10.7717/peerj.6257
Pytorch	Paszke et al.^41^	https://doi.org/10.48550/arXiv.1912.01703; RRID:SCR_018536
Python 3	Python Software Foundation	https://www.python.org; RRID:SCR_008394
SHAP	Lundberg and Lee^42^	https://github.com/slundberg/shap; RRID:SCR_021362

### Resource availability

#### Lead contact

Further information regarding this manuscript and requests should be directed to the lead contact, Vijaya B. Kolachalama, PhD (vkola@bu.edu).

#### Materials availability

This study did not generate any new materials.

### Method details

#### Study population and data selection

Data were collected from the Alzheimer’s Disease Neuroimaging Initiative (ADNI) for pre-training, training, internal validation, and internal testing (discovery dataset), and from the National Alzheimer’s Coordinating Center (NACC) for external testing (validation dataset).

#### ADNI cohort

ADNI comprises a longitudinal study consisting of data from many participating centers with an overall goal of facilitating the development of novel therapeutics by identifying biomarkers that identify AD and portend progression to AD. For this dataset, visits for all subjects were selected from the person registry with a last user date of April 9, 2020; this includes subjects enrolled in ADNI 1, ADNI GO, ADNI 2, and ADNI 3. General requirements for all phases of the study included persons between 55-90 years old, a partner able to be present for collateral, a Geriatric Depression Scale less than 6, and fluency in one of Spanish or English. Mild cognitive impairment (MCI) was defined by ADNI similarly across all 4 phases. To qualify as MCI, consistent criteria included the following: a person had to have 1) a complaint about some aspect of cognition; 2) Mini-Mental State Exam (MMSE) score ≥ 24 and a clinical dementia rating (CDR) equal to 0.5 with preserved daily function; and crucially, 3) some measured memory loss based on a Logical Memory test, adjusted for years of education. Persons had to have the amnestic domain affected to be enrolled. To meet criteria for AD, a person had to have a CDR ≥ 0.5, MMSE ≤ 26, an abnormal Logical Memory test, and meet criteria for AD based on NINCDS-ADRDA criteria for probable AD.^43^

Please see http://adni.loni.usc.edu/methods/documents/for more details.

From the collected data, the selected visit was defined based on when persons had a 3 Tesla T1-weighted MRI scan (ADNI search criteria included scans between 2.7 and 3.1 Tesla), CSF data collected, and a diagnosis of mild cognitive impairment (either late or early mild cognitive impairment where specified), as made by clinicians using multimodal criteria specified by ADNI. In sum, 51 persons were selected from ADNI1 (45 at the baseline visit, 2 at month 12, and 1 each at month 60, 96, 108, and 120), 113 from ADNIGO (112 at the baseline visit, 1 at month 24), 321 from ADNI2 (309 at the baseline visit, 11 at month 24, and 1 at month 48), and 59 from ADNI3 (all at baseline visit), yielding 544 total participants after having excluded 1 for poor image co-registration.

#### ADNI image selection

Raw MRI images on the ADNI database were queried using the keyword arguments ∗MP∗RAGE∗ and ∗SPGR∗, corresponding to magnetization-prepared rapid gradient-echo and spoiled gradient-recalled echo, respectively. Once downloaded, images were first filtered by the desired date for each person’s MCI visit. Then, the Mayo Clinic quality control information was used to further inform which image to use. If there was more than a single image at a given visit for each person, images were selectively kept using the following criteria in the following order of importance: being fully sampled (i.e., the image description did not contain the phrases “SENSE”, “ACCEL”, or “GRAPPA”); receiving a “pass” on the Mayo Clinic quality control sheet where this information was available; being taken at the most recent date; being chosen in the Mayo Clinic quality control sheet as “selected”; and finally, if there was still more than a single scan remaining, the image with the highest image ID, which generally corresponded to the latest image obtained in a series, was taken. If at any point application of these criteria led to removal of all scans for a given subject, the step was skipped to keep as many scans as possible (for example, if a subject only had accelerated MRI scans, accelerated MRI scans were used for that subject). This image selection process is illustrated in Figure S2.

#### CSF analysis

CSF data in the ADNI dataset were utilized from the UPENNBIOMK9 and UPENNBIOMK10 files provided on the ADNI website. In both files, biomarkers were analyzed via a Roche Elecsys e 601 instrument. Of note, the upper limit of this instrument was 1700 pg/mL, and the lower limit 200 pg/mL. Values above the upper limit were extrapolated via a calibration curve before they were downloaded from the ADNI website.

#### ADNI image curation for pre-training

For pre-training our deep-learning models, we constructed a dataset consisting of MRIs from unused persons in the ADNI cohort. These consisted of all 3-Tesla MRI images, IR-(F)SPGR and MP-RAGE, that we obtained at our collection date in the ADNI dataset, for persons that were not used for the main part of the study and did not at some point meet the criteria that included a diagnosis of MCI, CSF data, and a 3T MRI at a single visit. We sought to utilize images that were uncorrelated with the data that we were using for model evaluation and training, but that represented a type of data similar in quality to the training and testing data. Similar approaches were utilized previously with good success.^44^ Images that did not have diagnoses at the time of the visit were excluded, and we utilized images with a diagnosis of CN or AD for pre-training our final CNN model (Table 1).^44^ There were 4,827 total unique images. For three visits, two images existed carrying the same image ID. One of the images was selected and utilized twice in these cases.

#### NACC cohort

The NACC database hosts a Uniform DataSet comprised of longitudinal data, collected from persons in National Institute on Aging Alzheimer’s Disease Research Centers (ADRCs), each with its own protocol for enrollment, and each with its own protocol for diagnosis of disease (a team of physicians versus a single physician). Subjects in our study were selected from a data freeze on December 12, 2020. For each subject, visits where the subject had mild cognitive impairment (amnestic or non-amnestic, single, or multiple domain) were identified. In the NACC cohort, MCI persons were defined as those with preserved day to day function, though with a concern from the person, person’s partner, or physician about the person’s cognition and impairment in at least one cognitive domain. Dementia was diagnosed by a measured and clinically determined progressive decline in cognitive ability with impacted day-to-day function, in addition to impairment in at least one of five cognitive domains. AD was determined by clinical judgment based on available data.

Out of all visits for each person who had an MCI diagnosis, the visit closest to a date at which they had a 3T, T1-weighted MRI was kept. If the time between the clinical visit and MRI was longer than 6 months, the person was dropped from consideration. CSF values were assigned to the nearest diagnostic visit provided the visit occurred within ±6 months. CSF values in the NACC dataset were all obtained via an ELISA assay method (total of 21 samples).

Metadata for each of the T1-weighted scans were used to select which T1-weighted MRI to use out of the several MRIs available for each visit. Only three-dimensional, original, SPGR or MPRAGE images were used. Their single smallest dimension had to be at least 80 voxels. If a person had fully sampled scans, these were selected in place of any accelerated scans such as GRAPPA or SENSE. Finally, if there was more than a single image left for a person, an image collected that met the criteria was selected at random but with preference to the most recently acquired scans. Image curation and metadata pre-processing for NACC is shown in Figure S3.

#### Race and ethnicity

In terms of ethnicity, patients were classified as “Not Hispanic or Latino”, “Hispanic or Latino”, or “Unknown”. For our statistical analysis, patients with an “Unknown” ethnicity were excluded. For analysis of race, both cohorts contained the categories “Asian”, “Native Hawaiian or Pacific Islander”, “American Indian or Alaskan Native”, “White”, “Unknown”, and “Black or African American”. ADNI contained the additional designations “More Than One Race”, and NACC contained the additional designation “Multiracial”, which were considered to be the same. Several patients had different values for ethnicity at different visits, and several had different values for race at different visits. These patients were denoted as having a value of “Unknown” for the respective category. For the purposes of comparing proportions of non-white participants in either dataset, patients falling into the category “Unknown” were excluded.

#### Image registration, normalization, and segmentation

We utilized two pipelines for pre-processing MRI scans depending on the respective deep-learning model to be used. For models that required a single-dimensional input (the multi-layer perceptrons (MLPs), Cox proportional hazard (CPH) model, and Weibull model), we utilized the CAT12.7 v.1728 toolbox^40^ (https://neuro-jena.github.io/cat/) to parcellate brains into gray matter volumes (GMVs) and CSF volumes corresponding to the Neuromorphometrics atlas (Neuromorphometrics, Inc.). For our CPH model and base MLP, GMVs for each region of interest (ROI) were averaged across hemispheres and normalized to total intracranial volume, yielding hemisphere averaged, normalized GMVs. In addition, for these models, GMVs for regions corresponding to ventricles were removed from further analysis (CSF, 3rd, 4th, inferior lateral, and lateral ventricles). To align with the work of Nakagawa et al.*,*^13^ we kept laterality and ventricles in the Weibull model.

Separately, to ensure that excluding data regarding CSF volume and laterality did not significantly affect our MLP model fit, we constructed a supplementary multi-layer perceptron including normalized GMVs separately for each hemisphere, in addition to CSF volumes normalized by total intracranial volume separately for each hemisphere.

Regions were assigned to larger regions denoted as “lobes” as detailed in Table S4. Specifically, regions within the frontal, parietal, and occipital lobe were each grouped together. The temporal lobe was split into mesial and non-mesial temporal lobe, where the mesial temporal lobe consisted of the entorhinal area, parahippocampal gyrus, hippocampus, and amygdala. Other regions were assigned accordingly.

For our CNN model, which takes three-dimensional images as input, we constructed a pipeline to skull-strip raw T1-weighted MRIs and transform them into a common space. We used a standard SPM 12 v.7771 (https://www.fil.ion.ucl.ac.uk/spm/software/spm12/) pipeline consisting of (1) Segmentation of each brain into gray matter, white matter, and CSF and computation of normalization parameters; (2) Bias-correction of the initial brain; (3) Normalization of the bias-corrected brain into Montreal Neurological Institute (MNI) space; (4) Masking each normalized, bias-corrected brain by thresholding the sum of the gray matter, white matter, and CSF probability atlases at a value of 0.2, and taking the pointwise product of the normalized brain and the brain mask. Image processing steps are visualized in Figure S4.

#### CSF-based risk group analysis

##### Centroid computation

To ground our imaging findings in biomarker data, we utilized CSF amyloid-β (Aβ), which was widely available in the ADNI cohort. This is an attractive biomarker due to its direct implication early in the disease process. It is well established that Aβ level is related to rates of cognitive decline^45^ and can be used to identify persons with MCI who progress to AD.^46^ As a preliminary step, we divided MCI subjects in the ADNI cohort based on Aβ quartiles. GMVs for each person in ADNI were Z-scored region-wise using the respective mean and standard deviation for a given ROI, and Z-scored GMVs were averaged within each CSF-based risk group to compute centroids associated with each quartile of Aβ. These Z-scored GMV centroids were denoted as H (high), IH (intermediate-high), IL (intermediate-low), or L (low) risk, corresponding to the lowest through the highest concentration of CSF Aβ, respectively.

##### CSF-based risk group assignment

To assign final risk groups, GMVs for persons in ADNI and NACC were each Z-scored to the respective ROI’s mean and standard deviation from the ADNI dataset. Spearman’s correlation coefficient was then calculated between these Z-scored GMVs for each person and each of the four above mentioned centroids. The risk group corresponding to the highest correlation coefficient was assigned as the final risk group for that person.

#### Expert-driven assessment

For radiological analysis, a REDCap survey^47^^,^^48^ was administered to five independent, board-certified neuroradiologists in the United States. Forty-eight separate MRIs were selected, randomly, with 12 coming from each CSF-based risk group, 6 out of each 12 corresponding to persons who progressed from MCI to AD, and 6 out of each 12 who were censored or did not otherwise progress. Radiologists were first asked whether each of the following regions demonstrated atrophy in either hemisphere: frontal lobe, mesial temporal lobe, remainder of the temporal lobe, occipital lobe, parietal lobe, cingulate gyrus, and insula. If they identified the presence of atrophy, then they were asked to identify the extent to which each hemisphere demonstrated atrophy on a scale from no atrophy (assigned a numeric rating of 0) to severe atrophy (assigned a numeric rating of 3). Additional subregions within each lobe were assessed but not utilized for our final analysis.

#### Deep learning framework

All models were trained using the ADNI dataset. The data were split in a 3:1:1 fashion, and 5-fold cross validation was used. Models were tested on the NACC dataset. Pytorch was used for all deep learning analyses.^41^

##### Multi-layer perceptron

A multi-layer perceptron (MLP) was utilized to predict a person’s probability of progression from MCI to AD in each of three time bins following the MCI visit, using a survival loss function from Gensheimer and Narasimhan^22^:

$$Loss_{j}=\sum_{i}^{d_{j}}\ln\left({h_{j}}^{i}\right)+\sum_{i=d_{j}+1}^{r_{j}}\ln\left(1-{h_{j}}^{i}\right)$$

Where j stands for time interval j, ${h_{j}}^{i}$ is the disease hazard probability for individual i during time interval j (provided this individual didn’t progress yet), there are $r_{j}$ individuals ‘in view’ during the interval j (i.e., didn’t progress at the beginning of j), and the first $d_{j}$ of them progressed during this interval.

The total loss is then:

$$Loss_{S}=\sum_{j}^{N}Loss_{j}$$

Where Loss_S_ is the sum of loss for each time interval, and N is the total number of time intervals.

The time intervals are left-inclusive (i.e. [0,12), [12,24), etc.). Thus, when predicting that an individual, who is censored, survives at least to the end of interval j, the probability that they survive to that time is:

$$\mathrm{Pr}\left(Survival\right)=\prod_{i=1}^{j}\left(1-h_{i}\right)\text{.}$$

To compute the likelihood that a given interval progresses at time j, the formula is:

$$L=h_{j}\prod_{i=1}^{j-1}\left(1-h_{i}\right)$$

Finally, the likelihood of a given individual who is censored surviving to the latter half of time interval j-1 or the beginning half of j can be given as:

$$L=\prod_{i=1}^{j-1}\left(1-h_{i}\right)$$

For additional details, refer to Gensheimer and Narasimhan.^22^

Hemisphere-averaged, normalized GMV was utilized as input for this model as detailed above; that is, normalized GMV were averaged across hemispheres for each brain region (i.e., the right hippocampus and left hippocampus were averaged together to create a single hippocampus region).

The model architecture for this neural network consisted of a batch-normalization layer, followed by dropout, batch-normalization and leaky rectified linear-unit layers. This output was fed into a final linear layer, transformed via a sigmoid layer, and fed into a survival loss function. Thus, the network was trained to compute the *conditional probabilities of survival* in each of three time bins: 0-24 months, 24-48 months, and 48-108 months following the MCI visit, all left-side inclusive. The model was saved whenever it had a lower total survival loss on the validation set.

#### Survival convolutional neural network

A modified convolutional neural network (CNN) with a survival loss function was similarly trained to predict the risk of disease progression. T1-weighted MRI scans that were preprocessed with the SPM pipeline detailed above were used as input for this model. As a result of the different loss function, we denoted it as the survival convolutional neural network (S-CNN) (Figure S1). In this model, 3D convolution was used to handle the volumetric MRI scans. We performed hyperparameter optimization on the S-CNN model to maximize its potential. Specifically, we experimented with various combinations of layers (i.e., convolutional and dense layers, transformer and dense layers, etc.), different layer parameters (i.e., dropout rate, number of filters, batch size, learning rate, sample weights, and optimization metrics), as well as the use of transformed models from other datasets (i.e., fixed vs. learnable layers). During training, layers could range from 1 to 5; the dropout rate could vary from 0 to 0.75; the number of filters could vary between 1 to 50; the batch size was adjustable from 2 to 30; the learning rate could vary from 1e-8 to 1e-2; the sample weights included standard weight (where each sample carries the same weight) or inverse propensity weighting; the optimization metrics could be either loss-based or CI-based; and the final tuning of the transferred model involved freezing layers based on performance on the validation set. The final model was selected based on its concordance index at 24 months measured on the external (NACC) dataset.

For our final model, we utilized 2 convolutional layers and 2 dense layers. The model was trained for 2000 epochs, with a learning rate of 0.01, batch size of 10, drop rate of 0.3, filter number of 10 for first conv layer, and 20 for last conv layer. Stochastic gradient descent was utilized as an optimizer. Each convolutional layer’s kernel size was set to be 3, with a stride of 1 and no padding, and batch normalization was applied throughout the convolutional layers. After the normalization step, a leaky rectified linear unit was utilized as the activation function. Following this, a max-pooling layer of size 2 was attached. In addition to L2 normalization (weight=0.01), dropout layers (probability=0.3) were also used to boost the robustness of the network. Finally, we flattened the output and applied fully connected layers for the final prediction of risks. Before training, we initialized the weights using the default initializer (Kaiming Uniform method). Additionally, we calculated weights for each training sample based on their class frequency. During training on the MCI-to-AD prediction data, the model was saved whenever it had a better concordance index on the ADNI validation set at time 24 months.

Transfer learning is a method that initializes model A’s weights using a pretrained model B’s weights instead of random initialization. This method has been proven useful and is widely used in many tasks. We found that transfer learning improved the performance of our models. We adopt an approach similar to that of Oh et al.*,*^44^ and pre-trained our SCNN on a set of 4,827 images corresponding to persons diagnosed with either AD or normal cognition obtained from the ADNI dataset, except on a classification task using a cross-entropy loss. For final training, weights for all layers aside from the final dense layers were fixed. Parameters that differ from the above include a dropout rate of 0.01, learning rate of 0.001, and 100 training epochs.

#### Reference models

To compare our models against the current state-of-the-art, we trained a model based on Nakagawa et al.^13^ and an additional, more simple Cox regression with both L1 and L2 regularization.

Briefly, Nakagawa et al.^13^ use their training data to estimate parameters for a Weibull survival model. As input, they utilize parcellated gray matter volumes directly obtained using two atlases, the automated anatomical labeling atlas and the Brainnetome Atlas, via a similar process to ours using the Computational Anatomy Toolbox. Therefore, we utilized our raw parcellations from the Neuromorphometrics atlas, without averaging across hemispheres, as input to their model to establish a fair comparison. We constructed a model identical to theirs otherwise, though without early stopping and for 1000 total epochs instead of 200. Further, to accommodate the length of follow-up in our dataset, we extend predictions out to 9 years.

#### Cox proportional hazard model

Cox proportional hazard-based (CPH-based) models are used throughout the literature to estimate potential relationships between survival and a set of variables. Therefore, we constructed a baseline model using this framework utilizing the *python* package *lifelines*^49^ (https://lifelines.readthedocs.io/en/latest/). Breslow’s non-parametric method was used to estimate the baseline hazard function, and we utilized the same input used by our multi-layer perceptron model (hemisphere-averaged, normalized gray matter volumes) after Z-scoring these values to the training data for each fold. Models were trained using the same 3:1:1 cross-fold validation scheme, and grid-search was conducted utilizing the validation dataset to identify two parameters: the regularization weight, and the ratio of L1 to L2 loss. Concordance index was used as the metric of performance during grid-search. Survival predictions were obtained using the Cox Proportional Hazard class method *predict_survival_function*.

#### Interpretability analysis

##### SHAP value computation

SHaply Additive exPlanations (SHAP) were utilized to determine the contribution of each input feature (voxel) to the predicted survival of each person. SHAP values have been widely utilized to provide a measure of inference to machine learning models.^37^^,^^50^^,^^51^ To compute SHAP values, we utilized the DeepLIFT algorithm via the *deepexplainer* method from the SHAP package (https://github.com/slundberg/shap) in conjunction with our S-CNN model. Due to the large amount of memory required, a small subset of training data (6 samples) was used as the *background* data to compute expected values for each feature. SHAP values were computed for our NACC dataset and their absolute values were averaged across all three output time-bins (0-24, 24-48, and 48-108 months) and all five experiments for each person, yielding SHAP brains.

#### SHAP value analysis

For each SHAP brain, we first averaged across each risk group, generating 4 risk group averaged SHAP brains. We subtracted lower risk from higher risk group averaged SHAP brains, yielding subtracted SHAP brains. We then computed the mean and standard deviation across all voxels for each subtracted SHAP brain. The subtracted SHAP brains were Z-scored and thresholded at a value of 2.5 for plotting (Figure 5A).

Next, we sought to summarize the importance of each voxel to CNN output within brain regions. To achieve this task, we computed masks for each of 10 regions (basal ganglia, mesial temporal lobe, other temporal lobe regions, parietal lobe, cingulate gyrus, subcortical areas, occipital lobe, frontal lobe, cingulate cortex, and insula) using the Neuromorphometrics atlas obtained from SPM12 and computed the average, absolute voxel value for each SHAP brain within each of these regions. Pairwise comparisons were made within each risk group using pairwise Wilcoxon signed-rank tests, and p-values were corrected via a Benjamini-Hochberg procedure within each risk group. We plotted bar graphs of the mean absolute SHAP value, averaged across voxels within each lobe, using ggplot2, computing confidence intervals via a bootstrap method with 10,000 samples.

#### Pathology analysis

Postmortem pathology data were collated for persons in NACC to confirm accuracy of the model predictions. We used ADNC (AD neuropathological change) as an aggregate measurement of AD neuropathology, as it is a composite of characteristic amyloid plaques, neurofibrillary tangles, and neuritic plaques, the latter two of which translate to Braak staging and CERAD score, respectively.^52^ Persons were grouped by severity within each pathology measure and pairwise comparisons of proportions of persons who progress to AD were performed with Fisher exact tests with Benjamini-Hochberg correction for multiple comparisons.

### Quantification and statistical analysis

Throughout the manuscript, statistical significance was defined as a p-value less than 0.05 after correcting for multiple comparisons where necessary and as described below. Estimates for statistical parameters, degrees of freedom where applicable, and the *n* for each statistical test are included throughout the results section of the manuscript, aside from statistical tests involving comparisons for survival curves, where *n* are included in the respective figure (Figure 1 or Figure 2, respectively). Most statistical tests utilized in the manuscript are non-parametric (Kruskal-Wallis and Mann-Whitney U tests, for example), and therefore distributional assumptions were not required.

#### Demographics analysis

*Seaborn* (https://seaborn.pydata.org/) was used for scatter plots and violin plots in Figure 1,^53^ and *Scipy* (https://scipy.org/) was used to compute Kruskal-Wallis tests, Chi-square tests, and Mann-Whitney U tests in this figure.^54^ The p-values were corrected using Benjamini-Hochberg corrections (the Dunn test with a Benjamini-Hochberg correction is used for Kruskal-Wallis post-hocs). Bar plots were created using *pandas* version 1.0.1. (https://pandas.pydata.org/).

#### Gray matter volume analysis

To compare gray matter volumes between different CSF-based risk groups, Z-scored gray matter volumes were averaged across each brain region for each patient in the ADNI dataset. A Kruskal-Wallis test was performed using person-averaged, Z-scored gray matter volumes as individual datapoints.

#### Empirical survival analyses

For survival analyses, we utilized the *lifelines* package.^49^ The class KaplanMeierFitter was used to plot empirical Kaplan-Meier plots, and the function *survival_differences_at_fixed_point_in_time_test* was used to compute differences in survival at different time points. The CoxPHFitter class was used to compute hazard ratios and their 95% confidence intervals (Figures 1A and 2B). False discovery rate (FDR) p-value corrections were performed using a Benjamini-Hochberg correction for each family of pairwise p-values corresponding to each dataset and time point for Figure 2B for the *survival_differences_at_fixed_point_in_time_test*. For hazard ratios, FDR corrections were made for each family of pairwise p-values corresponding to each dataset. The *statsmodels* (https://www.statsmodels.org/stable/index.html) python library was used to compute FDR corrections.

#### Radiology analysis

For expert grading of MRI images, the order of the images was randomized in terms of CSF-driven risk-based subtype before being distributed to the radiologists. Grades for the cingulate cortex, frontal lobe, insula, mesial temporal lobe, non-mesial temporal lobe, occipital lobe, and parietal lobe were utilized. Radiologists also assessed atrophy of subregions within these areas, though these were not utilized for analysis for simplicity. First, radiology grades for each subject were averaged across both lobes and across radiologists and plotted using the *seaborn* package (Figure 3A). To compare between high and low-risk populations, the H and IH grades were treated as a single group and then tested against a group constituted by the L and IL grades. Mann-Whitney U tests were performed using the *statsmodels* package, with p-values corrected via a Benjamini-Hochberg procedure. To assess agreement in ratings between radiologists, pathology grades (none, mild, moderate, and severe) were converted into numeric scores, and intraclass correlation coefficients using absolute agreement were computed for each region in both hemispheres using the *ordinal* package in R. Intraclass correlation coefficients were chosen due to the ordinal nature of radiology grades.

Finally, to assess the relationship between gray matter volume in our parcellated atlas and radiologist grading, we obtained the average radiologist grading of each of the above brain regions and averaged these values between hemispheres and over all radiologists for each of the 48 individuals selected for grading. For comparison, gray matter volumes that were Z-scored using the mean and standard deviation of each region in the ADNI dataset were obtained. These values were averaged within each “lobe” as denoted in Table S4 and plotted against the radiologist gradings for each region using the *seaborn* package in python, with a 95% confidence interval computed using 1000 bootstrapped samples, the default in the function *lmplot* (Figure 3B). Pathology grades were converted into numeric scores as above. Spearman correlation coefficients were computed using *Scipy* and shown in Figure 3B.

#### Model-based statistics

Model performance was evaluated with concordance index (concordance_index_censored; CI) and integrated Brier score (integrated_brier_score; BS) for each of the test folds in our 5-fold cross validation scheme. The concordance index compares pairs of subjects and computes the proportion of pairs where our prediction of survival (i.e., which of the subjects “out-survives” the other) matches ground truth. We calculated the predicted probability of survival at 3 time points (i.e., bins), namely 24 months, 48 months, and 108 months. Concordance index was calculated at each time bin and averaged within each test fold. The Brier score is a statistic that measures the difference in survival for an individual against their predicted survival, and so measures the accuracy of our predictions. We utilized the training data for each fold to estimate the censoring probability at each time point. If the test data set contained datapoints outside of the range of the training data, predictions were truncated to this range and a new final bin survival probability was interpolated using a piecewise cubic Hermite interpolating polynomial (*Scipy*). These statistics were computed using scikit-survival (https://scikit-survival.readthedocs.io/en/stable/).^55^ Each model had a CI and BS for each of the 5 test folds. Statistical analysis was performed with pairwise paired Student T-tests and p-values were corrected with the Benjamini-Hochberg procedure.

#### Software packages

Some other software packages utilized were *colorcet* (https://colorcet.holoviz.org/, for certain color schemes), *lifelines*, *matplotlib* (https://matplotlib.org/), *nibabel* (https://nipy.org/nibabel/, for reading and writing Nifti files), *nilearn* (https://nilearn.github.io/stable/index.html, for plotting overlaid brain images), *numpy* (https://numpy.org/), *pandas*, *pytorch* version 1.7.0 or 1.9.0 (https://pytorch.org/), and *torchvision* version 0.8.1 or 0.11.3 (https://pytorch.org/vision/stable/index.html).

## Data Availability

• The raw data reported in this study cannot be deposited in a public repository as users must apply to either NACC or ADNI for access. Access to the raw imaging and demographic data are available via https://naccdata.org/ and http://adni.loni.usc.edu/ for the NACC and ADNI datasets, respectively. • All original code has been deposited at https://doi.org/10.5281/zenodo.8176269 and is publicly available as of the date of publication. • Any additional information required to reanalyze the data reported in this paper is available from the lead contact upon request. • Python scripts are made available on GitHub (https://github.com/vkola-lab/iscience2023).
