# Supplementary material for: Deep learning for risk-based stratification of cognitively impaired individuals
Source: iScience. 2023 Aug 2;26(9):107522. doi: 10.1016/j.isci.2023.107522 (PMC10460987; doi:10.1016/j.isci.2023.107522)
Supplement: Document S1. Figures S1–S4 and Tables S1–S4 [file mmc1.pdf]

iScience, Volume *26*

Supplemental information

Deep learning for risk-based stratification of cognitively impaired individuals

Michael F. Romano, Xiao Zhou, Akshara R. Balachandra, Michalina F. Jadick, Shangran Qiu, Diya A. Nijhawan, Prajakta S. Joshi, Shariq Mohammad, Peter H. Lee, Maximilian J. Smith, Aaron B. Paul, Asim Z. Mian, Juan E. Small, Sang P. Chin, Rhoda Au, and Vijaya B. Kolachalama

Figure S1. Survival convolutional neural network (S-CNN), related to Figure 4. Each convolutional layer (upper part) is composed by convolving and pooling operations, as well as dropouts. After 2 convolutional layers (middle part), the outputs were flattened and passed through two dense layers for final prediction. Before training, the network's parameters were initialized using a pre-trained CNN's weights for knowledge transfer (lower part), which was trained using inputs and labels corresponding to patients with either AD or normal cognition (NL). All layers aside from the final two dense layers were fixed following pre-training.

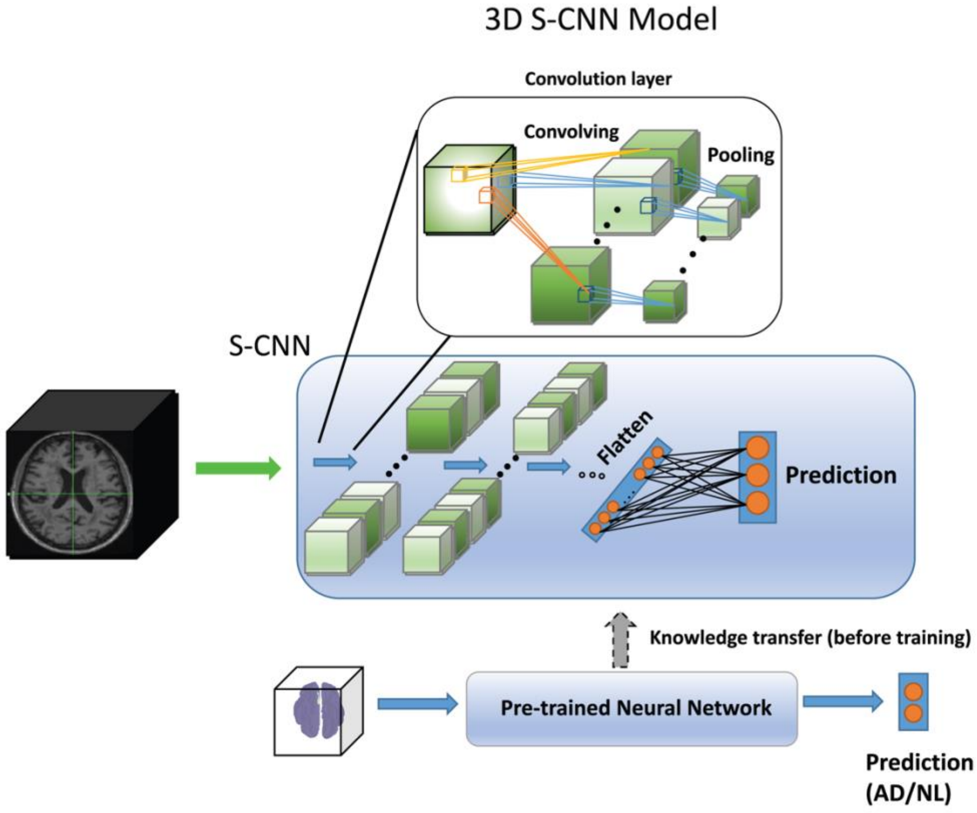

Figure S2. ADNI metadata curation, related to STAR Methods. A flow chart demonstrating the process of collecting and collating metadata from the ADNI cohort and aligning the metadata with images, produced via Lucidchart (lucid.app).

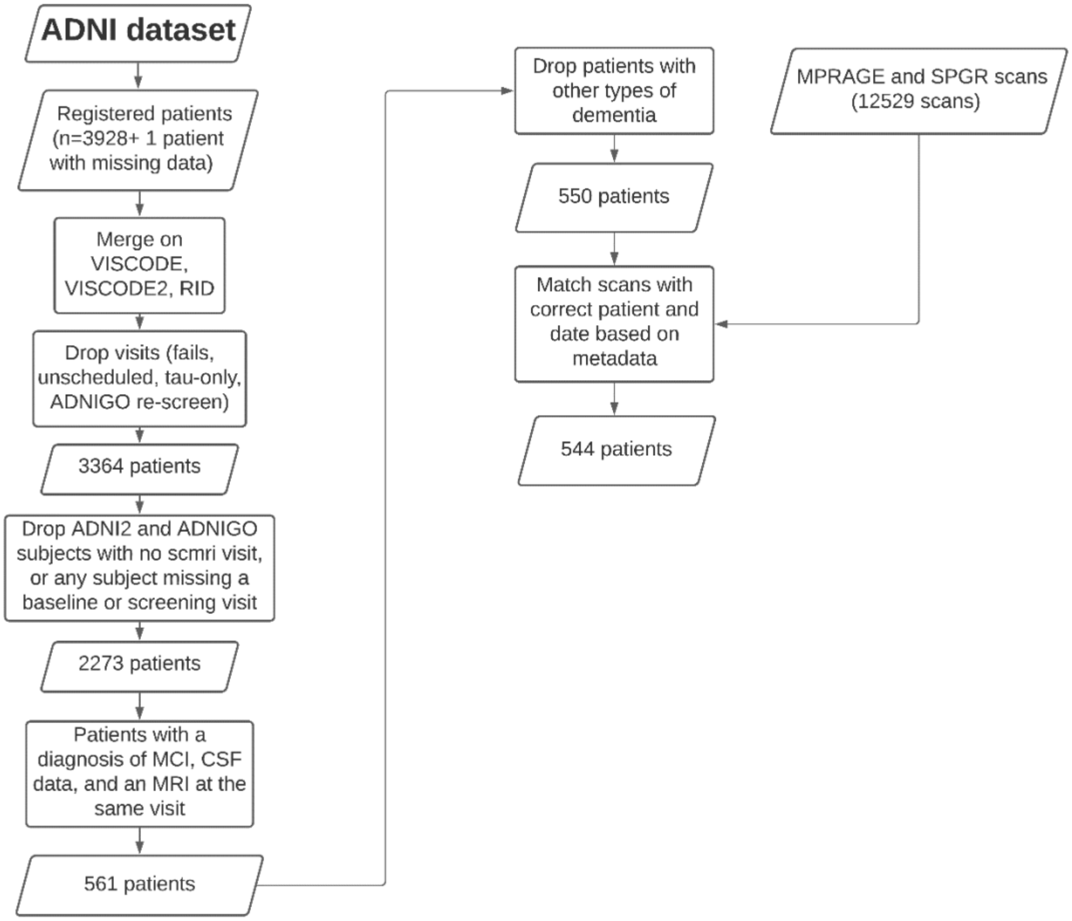

Figure S3. NACC metadata curation, related to STAR Methods. A flow chart demonstrating the process of collecting and collating metadata from the NACC cohort and aligning the metadata with images, also produced via Lucidchart (lucid.app).

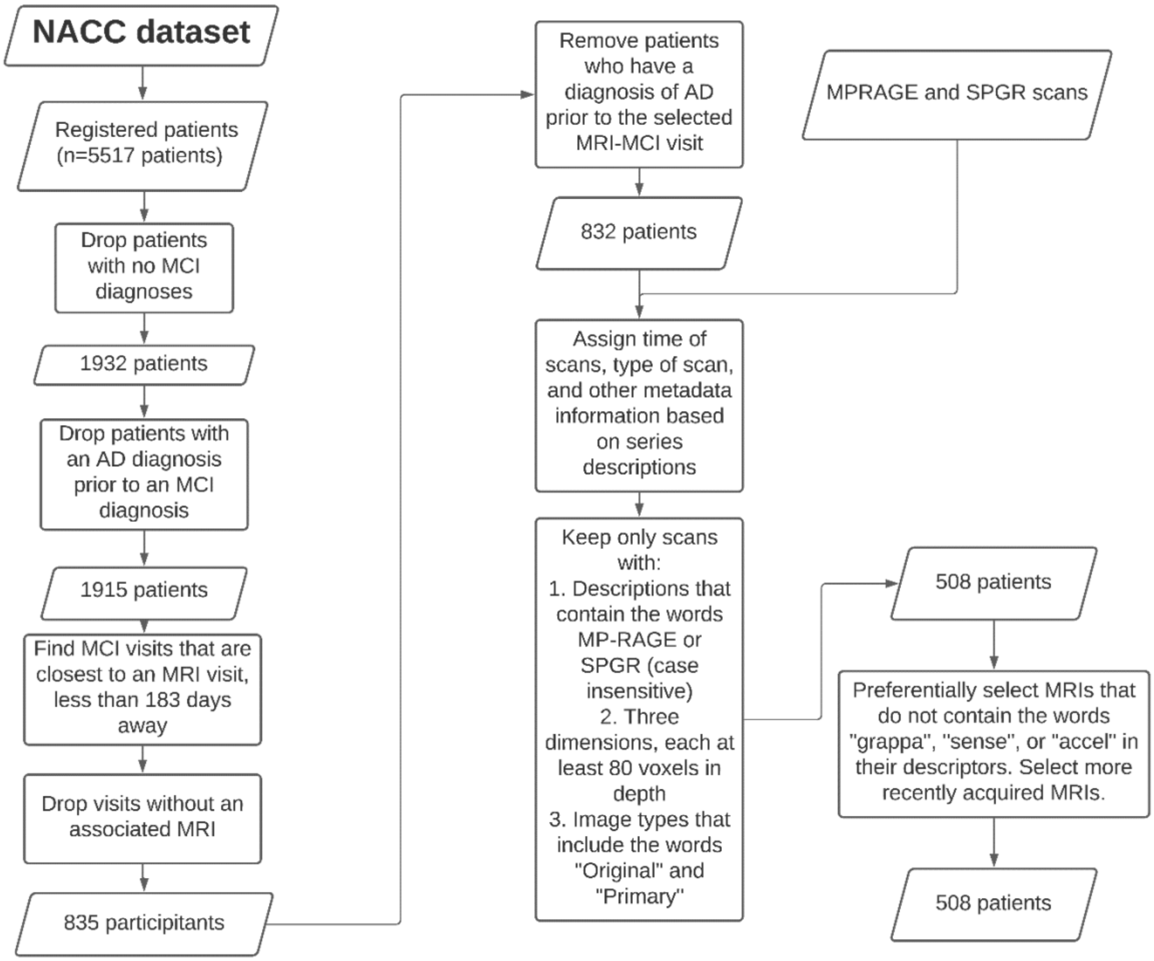

Figure S4. Image processing pipelines, related to STAR Methods. A flow chart demonstrating the two different image-processing pipelines. Given that our MLP required flat, parcellated input, we used a CAT12.7-based pipeline for MLP input due to the program's ease of use for this purpose. For the MLP, we obtained gray matter volumes corresponding to each region of the Neuromorphometrics atlas. Our CNN models required input in the form of three-dimensional MRI, so MRIs were processed with a simple SPM12 pipeline to bias-correct and skull-strip brains to be used by the S-CNN models. Also produced using Lucidchart (lucid.app).

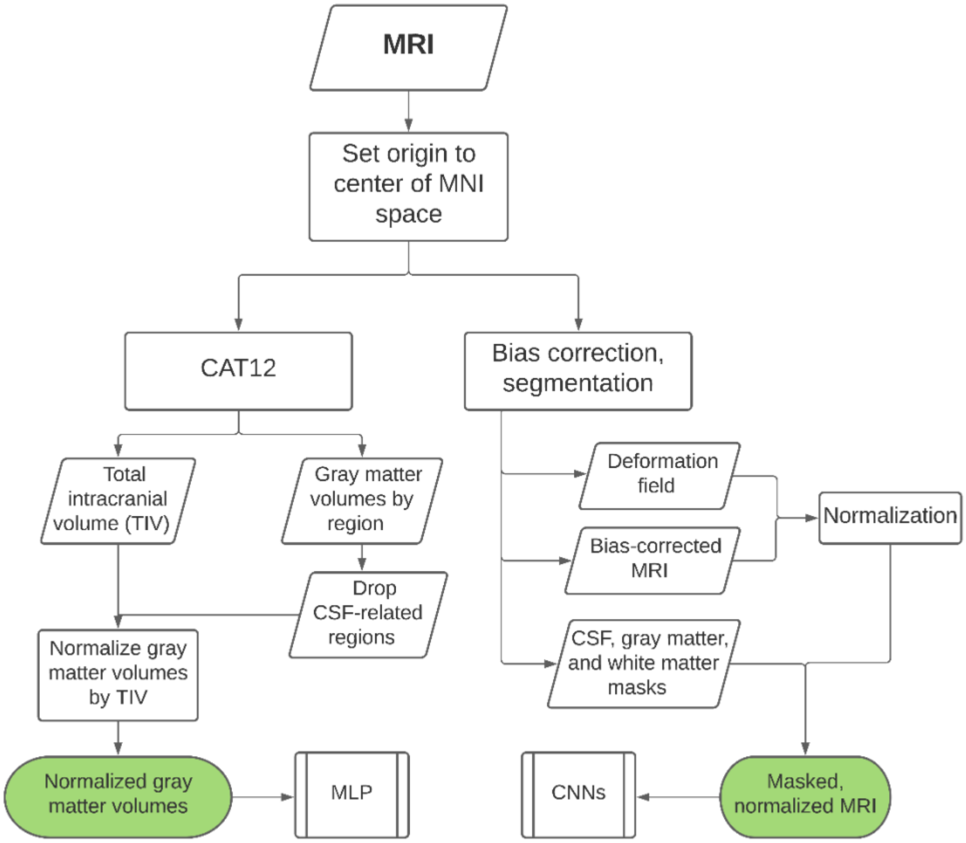

Table S1. Categorical demographic variables in Figure 1, related to Figure 1. Summary statistics for both the NACC and ADNI cohorts that correspond to categorical variables in Fig. 1. Ns are shown for each corresponding category. Data are divided into patients who progressed within different time frames (< 2 years, < 4 years, or ≥ 4 years), and into those who did not progress or were censored (Cens.).

Sex			<2 years	<4 years	≥4 years	Cens.	All
	NACC	Female	30	23	8	184	245
		Male	33	23	13	194	263
		All	63	46	21	378	508
	ADNI	Female	26	24	15	177	242
		Male	36	31	22	213	302
		All	62	55	37	390	544

APOE (# e4 alleles)							
	NACC	0 alleles	22	25	10	168	225
		1 allele	21	16	7	76	120
		2 alleles	9	3	4	18	34
		All	52	44	21	262	379
	ADNI	0 alleles	17	21	18	230	286
		1 allele	36	23	15	122	196
		2 alleles	9	11	4	38	62
		All	62	55	37	390	544

Table S2. Continuous demographic variables in Figure 1, related to Figure 1. Summary statistics for both the NACC and ADNI cohorts that correspond to continuous variables in Fig. 1. Medians, 25th and 75th percentiles are shown. Data are divided into patients who progressed within different time frames (< 2 years, < 4 years, or ≥ 4 years), and into those who did not progress or were censored (Cens.).

Age (years)			N	1st quartile	Median	3rd quartile
	NACC	<2 years	63	70.5	76	80
		<4 years	46	72.5	79	84
		≥4 years	21	73	78	84
		Cens.	378	65	73	78.75
	ADNI	<2 years	62	69.25	74	76
		<4 years	55	69.5	74	78
		≥4 years	37	69	75	79
		Cens.	390	66	71	77
MMSE	NACC	<2 years	33	24	25	28
		<4 years	34	26	27	28
		≥4 years	21	27	27	28
		Cens.	112	25	28	29
	ADNI	<2 years	62	25	26	28
		<4 years	55	26	27	29
		≥4 years	37	27	28	29
		Cens.	390	27	29	29
Education (Years)	NACC	<2 years	63	13	16	18
		<4 years	46	13	15	16
		≥4 years	21	14	16	18

		Cens.	378	14	16	18
	ADNI	<2 years	62	14	16	18
		<4 years	55	14	16	18
		≥4 years	37	14	16	19
		Cens.	390	14	16	18
Aβ42 (pg/mL)	NACC	<2 years	6	315	356	430.75
		<4 years	3	354	363	436.5
		≥4 years	3	359.5	419	531
		Cens.	9	436	476	674
	ADNI	<2 years	62	561.35	645.6	909.2
		<4 years	55	562.9	684.7	808.3
		≥4 years	37	568.6	779.8	1086
		Cens.	390	696.68	1057.5	1587
t-tau (pg/mL)	NACC	<2 years	6	570.75	611	772.75
		<4 years	3	475	676	927
		≥4 years	3	361.5	449	774.5
		Cens.	9	283	447	565
	ADNI	<2 years	62	257.63	328.65	456.6
		<4 years	55	265.3	340.7	453.9
		≥4 years	37	193.7	277.5	334.2
		Cens.	390	174.73	228.9	295.23
p-tau (pg/mL)	NACC	<2 years	6	59.75	62	63.5
		<4 years	3	48	64	102
		≥4 years	3	42.5	55	77
		Cens.	9	35	48	71
	ADNI	<2 years	62	23.99	32.41	45.89
		<4 years	55	25.27	34.01	49.29
		≥4 years	37	17.74	27.88	33.56
		Cens.	390	15.62	20.22	27.71

Table S3. Differences in survival between risk groups within each dataset at points in time, related to Figure 2.

	M	Comparison**	X^2	P	P_{BHC}*	N cens. (A)	N obs. (A)	N cens. (B)	N obs. (B)
ADNI	24	H – IH	7.024	0.0080	0.010	67	105	69	139
		H – IL	13.644	0.00022	0.00044	71	90	69	139

		IH – IL	1.719	0.19	0.19	71	90	67	105
		H – L	43.724	<0.0001	<0.0001	180	206	69	139
		IH – L	20.175	<0.0001	<0.0001	180	206	67	105
		IL – L	9.999	0.0016	0.0023	180	206	71	90
	48	H – IH	4.844	0.028	0.028	67	105	69	139
		H – IL	18.928	<0.0001	<0.0001	71	90	69	139
		IH – IL	5.588	0.018	0.027	71	90	67	105
		H – L	50.612	<0.0001	<0.0001	180	206	69	139
		IH – L	23.832	<0.0001	<0.0001	180	206	67	105
		IL – L	4.812	0.028	0.028	180	206	71	90
	96	H – IH	2.423	0.12	0.14	67	105	69	139
		H – IL	12.940	0.00032	0.0010	71	90	69	139
		IH – IL	5.963	0.015	0.022	71	90	67	105
		H – L	19.382	<0.0001	<0.0001	180	206	69	139
		IH – L	11.376	0.00074	0.0015	180	206	67	105
		IL – L	0.504	0.48	0.48	180	206	71	90
NACC	24	H – IH	2.957	0.086	0.13	100	129	77	134
		H – IL	7.503	0.0062	0.018	77	100	77	134
		IH – IL	1.822	0.18	0.21	77	100	100	129
		H – L	9.849	0.0017	0.010	124	145	77	134
		IH – L	3.001	0.083	0.128	124	145	100	129
		IL – L	0.110	0.74	0.74	124	145	77	100
	48	H – IH	9.488	0.0021	0.0062	100	129	77	134
		H – IL	4.709	0.030	0.060	77	100	77	134
		IH – IL	0.468	0.49	0.49	77	100	100	129
		H – L	13.803	0.00020	0.0012	124	145	77	134
		IH – L	1.039	0.31	0.37	124	145	100	129
		IL – L	2.534	0.11	0.17	124	145	77	100
	96	H – IH	7.792	0.0052	0.016	100	129	77	134
		H – IL	0.205	0.65	0.75	77	100	77	134
		IH – IL	2.271	0.13	0.20	77	100	100	129
		H – L	8.454	0.0036	0.016	124	145	77	134
		IH – L	0.104	0.75	0.75	124	145	100	129
		IL – L	2.848	0.092	0.18	124	145	77	100

* P_{BHC}=Benjamini-Hochberg corrected p-value
**H=high-risk group; IH=intermediate-high-risk group; IL=intermediate-low-risk group; L=low-risk group
Comparisons in survival curves corresponding to Fig. 2B. Pairwise-comparisons were made within each dataset and month (M column) between the probabilities of survival in each of the different risk groups (Comparison column). Survival and variance estimates were computed using Kaplan-Meier fits of either curve, and statistical tests were computed as described in the methods. P-values were adjusted via the Benjamini-Hochberg procedure (p_{BHC}). Degrees of freedom are 1 for this test.

Table S4. Region and “lobe" abbreviations and mappings used in throughout this manuscript, related to STAR Methods. Regions, their abbreviations, and their corresponding “lobe”. Region,

abbreviation, and region-to-abbreviation maps are as in the CAT12 package, and originate from: Neuromorphometrics, Inc. (neuromorphometrics.com/). Region-to-Lobe maps are modified from the same sources.

**Region Name**	**Abbreviation**	**Lobe**
3rd Ventricle	3thVen	
4th Ventricle	4thVen	
Accumbens	Acc	BG
Amygdala	Amy	TL-M
Angular Gyrus	AngGy	PL
Anterior Cingulate Gyrus	AntCinGy	Cing
Anterior Insula	AntIns	Ins
Anterior Orbital Gyrus	AntOrbGy	FL
Background	BG	
Basal Forebrain	BasCbr+FobBr	SC
Brainstem	Bst	
CSF	CSF	
Calcarine Cortex	Cal+Cbr	OL
Caudate	Cau	BG
Cerebellar Vermal Lobules I-V	CbeLoCbe1-5	
Cerebellar Vermal Lobules VI-VII	CbeLoCbe6-7	
Cerebellar Vermal Lobules VIII-X	CbeLoCbe8-10	
Cerebellum White Matter	CbeWM	
Supplementary Motor Cortex	Cbr+Mot	FL
Cerebral White Matter	CbrWM	SC
Central Operculum	CenOpe	FL
Cuneus	Cun	OL
Entorhinal Area	Ent	TL-M
Cerebellum Exterior	ExtCbe	
Frontal Operculum	FroOpe	FL
Frontal Pole	FroPo	FL
Fusiform Gyrus	FusGy	TL-O
Hippocampus	Hip	TL-M
Triangular Part of the Inferior Frontal Gyrus	InfFroAngGy	FL
Opercular Part of the Inferior Frontal Gyrus	InfFroGy	FL
Orbital Part of the Inferior Frontal Gyrus	InfFroOrbGy	FL
Inferior Lateral Ventricle	InfLatVen	
Inferior Occipital Gyrus	InfOccGy	OL
Inferior Temporal Gyrus	InfTemGy	TL-O
Lateral Orbital Gyrus	LatOrbGy	FL
Lateral Ventricle	LatVen	
Lingual Gyrus	LinGy	OL
Medial Frontal Cortex	MedFroCbr	FL

Medial Orbital Gyrus	MedOrbGy	FL
Postcentral Gyrus Medial Segment	MedPoCGy	PL
Precentral Gyrus Medial Segment	MedPrcGy	FL
Middle Cingulate Gyrus	MidCinGy	Cing
Middle Frontal Gyrus	MidFroGy	FL
Middle Occipital Gyrus	MidOccGy	OL
Middle Temporal Gyrus	MidTemGy	TL-O
Optic Chiasm	OC	
Occipital Fusiform Gyrus	OccFusGy	OL
Occipital Pole	OccPo	OL
Precuneus	PCu	PL
Pallidum	Pal	BG
Parahippocampus Gyrus	ParHipGy	TL-M
Parietal Operculum	ParOpe	FL
Planum Polare	Pla	TL-O
Postcentral Gyrus	PoCGy	PL
Posterior Cingulate Gyrus	PosCinGy	Cing
Posterior Insula	PosIns	Ins
Posterior Orbital Gyrus	PosOrbGy	FL
Precentral Gyrus	PrcGy	FL
Putamen	Put	BG
Gyrus Rectus	RecGy	FL
Subcallosal Area	SCA	FL
Superior Frontal Gyrus	SupFroGy	FL
Supramarginal Gyrus	SupMarGy	PL
Superior Frontal Gyrus Medial Segment	SupMedFroGy	FL
Superior Occipital Gyrus	SupOccGy	OL
Superior Parietal Lobule	SupParLo	PL
Superior Temporal Gyrus	SupTemGy	TL-O
Planum Temporale	Tem	TL-O
Temporal Pole	TemPo	TL-O
Transverse Temporal Gyrus	TemTraGy	TL-O
Thalamus Proper	ThaPro	SC
Ventral DC	VenVen	SC

*Lobes include OL – Occipital Lobe; FL – Frontal Lobe; TL-O – Temporal Lobe, non-medial; TL-M – Medial Temporal Lobe; BG – Basal Ganglia; PL – Parietal Lobe; SC – Subcortical regions; Ins – Insula; Cing – Cingulate Cortex
